# Hospital management of hyperglycemia in the context of COVID-19: evidence-based clinical considerations

**DOI:** 10.1186/s13098-022-00808-x

**Published:** 2022-03-04

**Authors:** Thiago Bosco Mendes, Alexandre Barbosa Câmara-de-Souza, Bruno Halpern

**Affiliations:** 1 Department of Internal Medicine, Hospital das Clínicas, São Paulo State University (UNESP), Botucatu, Brazil; 2 Department of Endocrinology, Hospital das Clínicas, University of São Paulo (USP), São Paulo, Brazil; 3 Department of Endocrinology, Hospital 9 de Julho, Rua Alves Guimarães, 462, cj. 72, Pinheiros, São Paulo, SP 05410-000 Brazil

**Keywords:** COVID-19, Hyperglycemia, Diabetes, Antihyperglycemic drugs, Corticosteroids, Insulin

## Abstract

The COVID-19 pandemic led to an unprecedented crisis, and early on, it has been shown that diabetes is an important risk factor for complications and mortality in infected patients, as demonstrated by several studies. Moreover, hyperglycemia, regardless of whether patients have diabetes, is associated with poorer outcomes, which suggests that adequate monitoring and treatment of elevated glycemia in the hospital setting can improve patient outcomes. In patients with COVID-19, glycemic control may be impaired as a consequence of the infection itself (aggravating pre-existing diabetes and potentially precipitating new-onset diabetes), inflammation, or corticosteroid use—a well-established therapy to reduce COVID-19 complications, especially in the intensive care unit. This article reviews the link between diabetes and hyperglycemia, and COVID-19, with a brief review of potential mechanisms, along with emerging evidence on the effect of glycemic control on COVID-19 outcomes, especially in hospital settings.

## Background

The COVID-19 pandemic struck the world in 2020, leading to an unprecedent crisis that urged the scientific community to rapidly understand the virus and the immune system response to it. Soon after the isolation of SARS-CoV-2 as the causative agent, diabetes proved to be an important risk factor for mortality in infected patients, which was proved by several epidemiological studies [1–7].

Before COVID-19, diabetes had already proved to be an important risk factor for adverse outcomes in different outbreaks. In the early 2000s, both diabetes and hyperglycemia were considered independent markers of death in individuals affected by SARS-CoV-1 [8]. Further on, in the next decade, patients with diabetes and Middle East Respiratory Syndrome (MERS) also had poorer outcomes [9]. Notably, COVID-19 can lead to an inflammatory storm and it has been postulated that type 2 diabetes (T2D) proinflammatory features contribute to its development [10].

It is also important to highlight that hyperglycemia and COVID-19 may have a multidirectional relationship. Since the virus binds to angiotensin-converting enzyme 2 (ACE2), which is widely expressed and present on pancreatic beta cells and adipose tissue, it is very likely that SARS-CoV-2 can, by itself, impair glucose metabolism [11]. It has already been shown that SARS-CoV-2 infects endocrine and exocrine pancreatic cells that are close to the islet of Langerhans [12]. Indeed, an in vitro study demonstrated that SARS-CoV-2 reduces pancreatic insulin secretion and induces β-cell apoptosis in a manner that resembles what is observed in type 1 diabetes (T1D) [13]. Given that T1D incidence has risen recently, there is also a concern that COVID-19 is a potential risk factor for T1D [14–16], which is still a matter of debate given the possibility that other factors have driven this increase.

In the adipose tissue, a study assessing hamsters infected by SARS-CoV-2 found reduced adiponectin expression, confirming that the infection can change adipocytes metabolism, potentially driving insulin resistance [17].

In the inpatient setting, it is well known that the diagnosis and treatment of hyperglycemia are of paramount importance and reduce morbidity, length of stay, intensive care unit (ICU) need, and even in-hospital mortality. Failure to recognize and treat hyperglycemia leads to an increase of up to 5.8 times in the risk of hospital infection after surgery [18], worsened functional recovery after stroke [19], worsened outcomes for patients with myocardial infarction [20], and increased risk of thrombotic events [21]. The negative impact of hyperglycemia in hospitalized patients is greater in those without a previous diabetes diagnosis, who have an in-hospital mortality rate almost ten times higher than normoglycemic patients [22].

Moreover, glucocorticoids (GCs) use for the treatment of COVID-19 was studied in multiple randomized controlled trials (RCTs) and a meta-analysis of RCTs in the treatment of COVID-19, showing a substantial benefit in COVID-19 patients with hypoxemia, including mortality reduction [23–27]. Although the benefits of GC in COVID-19 are well described and replicated in several studies, it is unclear if these benefits are similar in individuals with different baseline comorbidities. Both GC and severe COVID-19 impair glucose metabolism [28–31], making it imperative to detect and treat hyperglycemia in these patients.

This article intends to provide evidence linking hyperglycemia with COVID-19, giving a brief review of potential mechanisms, and justifying the importance of glycemic control as a major treatment strategy in hospitalized patients.

## Diabetes, hyperglycemia and COVID-19 outcomes

The English OpenSAFELY initiative, which evaluated thousands of deaths from COVID-19, found a hazard ratio of 1.31 (CI 95%: 1.24–1.37) for death in patients with T2D and glycated hemoglobin (A1c) < 7.5%, and 1.95 (CI 95%: 1.82–2.08) for patients with T2D and A1c ≥ 7.5% compared to those without T2D, after adjusting for age, sex, ethnicity, and multiple comorbidities [2]. It also illustrates the importance of measuring A1c levels in hospitalized patients with hyperglycemia and no previous diagnosis of diabetes, which helps to differentiate patients with previously unrecognized T2D (A1c ≥ 6.5%) from those with stress-induced hyperglycemia [32]. Although both situations have been associated with increased mortality due to COVID-19, stress-related hyperglycemia in the COVID-19 setting is likely worse [5, 6], with a mortality of 41.7% compared to 14.8% in those with diabetes [5].

In a retrospective analysis, Zhu et al. observed that, in patients with T2D, blood glucose (BG) control, defined as ≤ 180 mg/dL (10 mmol/L), during hospitalization for treatment of COVID-19 was associated with a hazard ratio of 0.14 (confidence interval [CI] 95% 0.03–0.60) for mortality after matching using a propensity score modeled for age, gender, hospital, severity of COVID-19, and several comorbidities [4].

*Bode et al.* analyzed 570 patients with outcomes from COVID-19 admitted to 88 US hospitals. The mortality rate for the combined population with diabetes or uncontrolled hyperglycemia during hospitalization was 28.8% *vs* 6.2% for those without diabetes and hyperglycemia during hospitalization [5].

Another study in China assessing 453 patients showed that newly diagnosed diabetes during hospitalization for COVID-19 treatment, defined as A1c ≥ 6.5% and/or fasting glucose ≥ 126 mg/dL (7 mmol/L), was associated with increased mortality risk [6]. Also, a Spanish study showed that hyperglycemia at admission in patients hospitalized due to COVID-19 is a predictor of mortality [33].

Patients with type 1 diabetes (T1D) have an increased risk of hospitalization due to COVID-19 across all age groups compared to those without diabetes [34]. An extensive analysis evaluating 23,698 COVID-19 related deaths in England showed mortality rates per 100,000 habitants over a 72-day period to be 27 for those without diabetes, 138 for those with T1D and 260 for those with T2D [1].

Although several clinical studies as cited above indicate an important association between hyperglycemia and worsened outcomes, it is essential to emphasize that confounders related to illness severity, and SARS-CoV-2 itself, leading to hyperglycemia, may account for at least a proportion of this association.

## Potential mechanisms for hyperglycemia-caused worse COVID-19 outcomes

There are several hypothesized mechanisms by which hyperglycemia can impair response to infections leading to poorer outcomes, such as a reduction in neutrophil degranulation, impairment of complement activation, and impairment in phagocytosis [35].

Hyperglycemia results in the activation of nuclear factor kB, production of proinflammatory cytokines and oxidative stress that increase endothelial dysfunction [36]. Although the humoral immune response to SARS-CoV-2 is not broadly impaired in patients with diabetes, more data is needed to better understand the cellular response in patients with diabetes infected by the virus [37], even though it is already known that diabetes is a proinflammatory disease that potentially decreases interferon response [38].

Specifically for SARS-CoV-2, elevated glucose directly promotes viral replication and cytokine production, with subsequent T cell dysfunction and lung epithelial cell death [35]. High glucose levels can also occur in pulmonary secretions and possibly increase the rate of virus infection and replication, as is the case for the influenza virus [39].

Insulin resistance (IR) per se can also be a risk factor for infections. A recent review postulated that IR increases cytokine production, alters adipose tissue hormones (decreased adiponectin), increases C3, increases microthrombosis, impairs systemic microcirculation, and increases ectopic lipids in the alveolus. As such, modest caloric restriction and weight loss can have profound effects [40, 41]. An ecological analysis using multivariable linear regression to evaluate differences in COVID-19 deaths in 30 countries found obesity to be the strongest factor associated with death (correlation of 0.297) [42]. It is possible, but not clear, that IR is an independent risk factor for severer infection outcomes. If this is the case, hyperglycemia correction can only partially reduce the increased risk for worsened outcomes in T2D patients, and other strategies, such as weight loss, can potentially lead to better outcomes. Nonetheless, the impact of weight loss in the acute setting is unknown. Patients with diabetes also have more comorbidities, such as hypertension and cardiovascular disease, that worsen COVID-19 outcomes [43].

In summary, hyperglycemia may impair several different steps of the immune response and may also facilitate virus replication. An extensive review of potential mechanisms is beyond the scope of this manuscript and can be found elsewhere [35–39].

## Hyperglycemia and diabetes in hospital settings in the context of COVID-19

It is recommended that all patients admitted to a hospital should be questioned about a history of diabetes. Importantly, point-of-care glucose should be evaluated at admission of any patient, regardless of a previous history of diabetes and glycemic monitoring should be provided to those at risk for hyperglycemia during hospitalization [44]. Hospitalized patients with T2D or transient stress hyperglycemia, defined as fasting BG > 140 mg/dL (7.8 mmol/L) or any glucose value ≥ 180 mg/dL (10 mmol/L), while receiving GCs should test their glucose levels at least four times a day: before meals and at bedtime [45]; or, if the patient is fasting, every 4–6 h [46]. In addition, it has been suggested that in-hospital patients with COVID-19 should have their glucose monitored at least four times a day for at least 48 h, even if they are euglycemic at admission [47].

Patients with established diabetes [44] or BG ≥ 180 mg/dL (10 mmol/L) [32] require therapeutic intervention to control hyperglycemia. The American Diabetes Association (ADA) also recommends that all patients with known diabetes or in-hospital hyperglycemia should have their A1c levels tested if this has not been done in the preceding two to three months [32].

### Glycemic targets in hospitalized patients with COVID-19

The ADA recommends a BG target range of 140–180 mg/dL (7.8–10 mmol/L) in hospitalized patients to avoid both hyperglycemia and hypoglycemia, regardless of the cause for admission. Generally, it has been considered that more relaxed control targets should be pursued for elderly people with a high risk of hypoglycemia. Thus, a BG ≥ 180 mg/dL (10 mmol/L) may be acceptable in the inpatient care setting, especially in situations in which frequent glucose monitoring and close nursing supervision is not feasible [32].

Concerning COVID-19, a small (n = 25) non-randomized controlled trial showed that glycemic control with a mean glycemia of 138 mg/dL (7.7 mmol/L) compared to 192 mg/dL (10.7 mmol/L) for patients with hyperglycemia was associated with a significant reduction in a composite endpoint including intensive care admission, mechanical ventilation, or death (33% *vs* 80%) [48]. In a retrospective assessment using data from a software that titrates insulin doses in 91 US hospitals, non-ICU patients hospitalized due to COVID-19 that had hyperglycemia > 250 mg/dL (13.9 mmol/L) in the 2–3 days after hospitalization had an adjusted estimated hazard ratio of 7.17 (CI 95%: 2.62–19.62) for mortality compared to patients with BG < 140 mg/dL (7.8 mmol/L) in a multivariate analysis [49]. Another study observed that, in patients with T2D, BG ≤ 180 mg/dL (10 mmol/L) during hospitalization for treatment of COVID-19 was associated with a hazard ratio of 0.14 (CI 95%: 0.02–0.60) for mortality compared to BG > 180 mg/dL (10 mmol/L) after matching using a propensity score modeled for age, gender, COVID-19 severity, and comorbidities [4].

### Therapeutic strategies in hospitalized patients with T2D or no previous diabetes

Insulin is the first-choice treatment for glycemic control given its efficacy and the possibility of quick titration. An intensive regimen with subcutaneous basal and prandial insulin is the best treatment for non-critically ill hospitalized patients [32, 50, 51]. The initial total daily dose of insulin recommended to avoid both hypoglycemia and hyperglycemia is 0.4–0.6 IU/kg of body weight [52], but may be lower, at 0.2 UI–0.3 IU/Kg of body weight, in patients aged over 70 years old or with an estimated creatinine clearance below 60 mL/min [44]. The total daily dose of insulin should be distributed as 50% of basal insulin and 50% of short-or-rapid insulin equally divided before each of the three main meals [44]. Notably, insulin requirement varies from person to person and patients taking GCs may require higher doses in the range of 1.2 to 1.5 IU/kg daily [53].

Basal insulin only or basal plus bolus regimens are preferred in noncritically ill hospitalized patients with poor oral intake [32]. Even if the patient is fasting, the basal insulin should not be withheld. A comprehensive table for prandial insulin dose adjustment according to BG can be found elsewhere [44]. Of note, sliding-scale insulin as the only regimen is not recommended [32] as it is a reactive method that provides insulin only after hyperglycemia.

### Therapeutic strategies in hospitalized patients with T1D

All T1D patients should be treated with insulin. For those who had been receiving mixed insulin at home, changing to a basal bolus regimen is recommended as it reduces hypoglycemia risk [54]. Decreasing the usual at home rapid insulin dose by 20–30%, especially in those who have decreased appetite, decreases the risk of hypoglycemia. For insulin naïve patients, the initial total daily dose of insulin should be between 0.3 and 0.6 IU/kg, depending on hyperglycemia severity. The total daily dose of insulin should be distributed as 50% of basal insulin and 50% of short-or-rapid insulin equally divided before each of the three main meals [54].

More intensive BG monitoring and supportive therapy to reduce the risk of metabolic decompensation leading to events such as diabetic ketoacidosis (DKA) or hypoglycemia is required [55]. Additionally, blood or urinary ketones should be checked if hyperglycemia or fever develop [53].

### Therapeutic strategies in critically ill patients

Insulin is the most adequate therapy for glucose control in severely ill patients, and hypoglycemia risk should be addressed [32]. Increased vigilance, detection, treatment, and close monitoring of glucose should be implemented to avoid unfavorable clinical outcomes. Even in patients with T2D, intravenous infusion based on validated local protocols and adjusted according to BG until stable glycemic control is achieved should be pursued [56].

Among critically ill patients on intravenous insulin infusion, BG should be monitored every 1–2 h. After BG values are stable and on the target range, BG can be checked in longer intervals, such as every 4 h [57].

One of the biggest challenges for healthcare systems is to manage glucose in severely ill patients who are in isolation, especially with limited staff and personal protective equipment. The ADA suggest that establishing protocols and structured orders sets using computerized support may help to improve BG control [32].

A significant concern in severely ill patients with diabetes and COVID-19 is secondary bacterial infection. For this reason, monitoring parameters that indicate worsening inflammation such as ferritin, complete blood count, and C-reactive protein may be of benefit [47]. Several agents targeting inflammation are being tested as treatment for COVID-19 [58, 59], but it is not the scope of this review to discuss treatments beyond hyperglycemia.

### Non-insulin medications

Non-insulin hypoglycemic agents are generally not recommended for hospitalized patients, but some medications have been studied and can be considered in specific situations in patients with COVID-19 and T2D [56]. Particularly in those with not severe COVID-19, oral agents can be used in hospitalized patients with T2D, as long as there is no contraindication and they do not cause hypoglycemia [60]. Of note, it is unanimous that insulin should be maintained for patients with T1D or those with T2D on insulin at home. Furthermore, acute illnesses, such as COVID-19, may require a higher amount of insulin [61].

Theoretically, some hypoglycemic agents such as glucagon-like peptide-1 receptor agonists (GLP-1RA), dipeptidyl peptidase-4 inhibitors (DPP4i) and a thiazolidinedione (pioglitazone) can improve outcomes in patients with COVID-19 because of their anti-inflammatory effects. Indeed, a multinational retrospective analysis of non-hospitalized patients with COVID-19 adjusted for several confounders showed that continuing these medications was associated with better outcomes compared to stopping these drugs [62]. As this data is observational, not in hospitalized patients, and has not been confirmed, care is warranted to avoid over-interpretation of these data.

The DARE-19 was the first trial to randomize hospitalized patients with COVID-19 for an oral T2D medication. Dapagliflozin or placebo was given to hospitalized COVID-19 patients with at least one cardiometabolic risk factor, meaning that both patients with and without diabetes were eligible. Despite being well tolerated, dapagliflozin did not reduce the primary composite endpoint of organ dysfunction or death. Only two non-severe events of diabetes ketoacidosis (DKA) were reported in the population that received dapagliflozin, even with rigid surveillance [63], which is reassuring. Prior to this study, many experts discouraged the use of sodium/glucose cotransporter-2 inhibitors (SGLT2i) in patients with severe disease due to the increased risk of DKA [64]. As this new data emerged, continued use of this drug if DKA is monitored is a possibility. Also important is a trend towards better outcomes with dapagliflozin. Given that the total number of primary outcome events was lower than expected, and there was a statistically nonsignificant 20% reduction in the primary outcome in the group receiving dapagliflozin, some experts advocated this study was underpowered [63, 65, 66]. The RECOVERY group announced a larger trial with empagliflozin to further evaluate the hypothesis that SGLT2i can be of benefit in this setting [67]. Importantly, this drug class is contra-indicated in the setting of acute disease in patients with T1D due to the increased risk of DKA [68, 69].

Before COVID-19, several RCTs demonstrated the efficacy and safety of using GLP-1 RA and DPP4i in specific groups of hospitalized patients. This strategy is an option, but it is important to mention that the discontinuation of alogliptin and saxagliptin is recommended for patients with heart failure [32].

Metformin treatment prior to COVID-19 diagnosis was independently associated with a significant reduction in mortality in patients with diabetes and COVID-19 (odds ratio: 0.33, 95% CI: 0.13–0.84) in a retrospective assessment [70]. Even tough lactic acidosis is a concern for hospitalized patients taking metformin, especially with acute illnesses, such as COVID-19 [61], continuing this drug may be of benefit for hospitalized COVID-19 patients [53].

In summary, non-insulin hypoglycemic agents are not the first-line treatment for hyperglycemia in hospitalized patients, but these drugs are being successfully used in patients hospitalized due to COVID-19. Maintaining treatment with hypoglycemic agents used at home should be considered, especially in patients with moderate, not severe, COVID-19.

### Considerations on specific situations

#### Glucocorticoids

Considering the benefit of GCs in patients with respiratory failure due to COVID-19 [23–27], there is a need for specific insulin therapy to address steroid-induced hyperglycemia. GCs potentially aggravate hyperglycemia in patients with diabetes, trigger symptoms in patients with undiagnosed diabetes or precipitate GC-induced diabetes [71].

Before COVID-19, a study found that 10% of hospitalized patients receiving GCs do not have medical orders for BG [72]. Monitoring BG in these patients is necessary. Of note, GC-induced hyperglycemia often occurs in the late afternoon and evening.

#### Diabetes-associated emergencies in COVID-19 patients

COVID-19 has been linked to both DKA and hyperosmolar hyperglycemic state (HHS). The pathophysiology of this increased risk is yet to be fully determined, but contributing factors are β cell damage caused by the virus and increased inflammation [73]. One study assessing 175 hospitals found the mortality due to DKA in patients with COVID-19 to be higher across all age groups compared to that of DKA in patients without COVID-19 [74].

In hyperglycemic emergencies, temporary intravenous insulin pump use with continuous insulin infusion coordinated by a local diabetes inpatient team should be done when possible [32]. There is, however, a valid concern that the continuous insulin infusion, which requires greater patient attention by healthcare workers, increases these workers’ exposure to the virus. For these reasons, a guidance for DKA management with rapid-acting subcutaneous insulin every four hours and an initial dose of 0.4 IU/kg/4 h was developed and is freely available [75]. There is also a report in which a patient with COVID-19 and DKA was successfully treated with insulin glargine 0.15 IU /kg and insulin aspart 0.3 IU/kg as a loading dose, followed by insulin aspart 0.2 IU/kg every four hours until DKA resolution [76].

#### Flash glucose monitoring

Flash glucose monitoring offers the advantage of reducing healthcare workers exposure to patients infected by SARS-CoV-2, an approach that was recognized by the FDA—Food and Drug Administration [77]. In the COVID-19 era, a hospital in Colombia used it and provided a 72.5% median time of glucose in range [78]. However, this is an expensive method that requires healthcare workers training and caution regarding interferents in the readings. These interferents, such as hypoxemia and paracetamol are often present in patients with COVID-19. If this method is used, a reasonable goal is to maintain glucose within range at least 70% of the time [61, 79].

#### Transition from hospital to home

For patients who have an A1c ≲ 7%, the regimen before hospitalization should be continued after discharge. If the A1c is > 7% without classic diabetes symptoms, intensifying the treatment by associating another drug is recommended. For those with A1c > 7% and symptomatic hyperglycemia, it is recommended to start or increase the dose of insulin at least one day before discharge to check both the effectiveness and safety of the new strategy [44].

## Conclusion

Healthcare professionals must know the risks of hyperglycemia in hospitalized patients with COVID-19 and its treatment. Frequent assessment of BG, rapid recognition of DKA, and glycemic control eventually save lives. Protocols designed according to each hospital’s structure are also welcomed. Nonetheless, strict glucose control benefits should be balanced against the risk of severe hypoglycemia in hospitals where intensive blood glucose monitoring and nursing surveillance are not feasible, especially in the scenario of a shortage of personnel and supplies.

Observational studies clearly show that glycemic control using insulin decreases the rate of important undesired outcomes in patients with COVID-19, such as mechanical ventilation, ICU admission, and death. Interestingly, for patients hospitalized because of COVID-19, oral antihyperglycemic drugs are being successfully used, especially in those who are already taking the medication regularly before being infected and do not have severe hyperglycemia. The dapagliflozin RCT showed that the drug does not add a benefit itself to the COVID-19 treatment arsenal in the hospital, although it is safe in this setting. By now, insulin is still the gold standard for in-hospital glycemic control, regardless of the cause for hospitalization.

## Data Availability

Not applicable.
